# Affective Polarization in Comparative and Longitudinal Perspective

**DOI:** 10.1093/poq/nfad004

**Published:** 2023-02-24

**Authors:** Diego Garzia, Frederico Ferreira da Silva, Simon Maye

**Affiliations:** Assistant Professor, Institute of Political Studies, University of Lausanne, Lausanne, Switzerland; Senior Researcher, Institute of Political Studies, University of Lausanne, Lausanne, Switzerland; Research Assistant, Institute of Political Studies, University of Lausanne, Lausanne, Switzerland

## Abstract

Existent research shows that affective polarization has been intensifying in some publics, diminishing in others, and remaining stable in most. We contribute to this debate by providing the most encompassing comparative and longitudinal account of affective polarization so far. We resort to a newly assembled dataset able to track partisan affect, with varying time series, in eighteen democracies over the last six decades. We present results based on two different operational measures of affective polarization: Reiljan's Affective Polarization Index, based on reported partisans only, and Wagner's weighted distance from the most liked party, based on the whole electorate. Our reassessment of affective polarization among partisans confirms that an intensifying trend is observable in a number of countries but it is, by no means, generalizable to all established democracies. Regarding the longitudinal assessment of affective polarization among the electorate, we confirm that US citizens have become more affectively polarized over time.

## Introduction

Initial scholarship on polarization originated in the United States and focused largely—if not exclusively—on policy preferences (Iyengar, Sood, and Lelkes 2012; Lauka, McCoy, and Firat 2018). More recently, a novel perspective based on social identity theory has brought forward the notion of *affective* polarization, namely, “the extent to which partisans view each other as a disliked out-group” (Iyengar, Sood, and Lelkes 2012, p. 406).

A growing body of evidence shows that affective polarization is indeed on the rise in the United States. This development appears to be driven by voters’ tendency to increasingly dislike parties and candidates they do not support. Available research shows that US voters’ evaluation of their own parties and candidates is stable, yet they have come to dislike their opponents more over time (Iyengar, Sood, and Lelkes 2012, p. 413; Abramowitz and Webster 2016, p. 15; Finkel et al. 2020, p. 534).

Comparative research going beyond American affective polarization has emerged in recent years (Gidron, Adams, and Horne 2020; Reiljan 2020; Boxell, Gentzkow, and Shapiro 2021; Garzia and Ferreira da Silva 2021; Wagner 2021). These studies converge on the finding that levels of affective polarization are not especially prominent in the United States, but that polarization has grown stronger there than anywhere else. However, the picture remains unclear when it comes to other countries. Gidron, Adams, and Horne (2020) analyze data from the Comparative Study of Electoral Systems (CSES) for 20 democracies over the 1996–2017 period and conclude that America’s intensifying affective polarization is *not* part of a cross-national trend. Instead, they find that affective polarization has been “intensifying in some publics, diminishing in others, and remaining stable in most” (p. 8). Boxell, Gentzkow, and Shapiro’s (2021) analysis of twelve established democracies extends the window of observation back to the late 1970s, and concurs that “the US stands out for the pace of the long-term increase in affective polarization” (p. 3).

We contribute to this debate by further extending the number of countries and the time span under analysis, providing the most encompassing comparative and longitudinal account of affective polarization so far. We do so by resorting to a newly assembled dataset able to track partisan affect, with varying time series, in eighteen established democracies over the period 1961–2020. Moreover, we present results based on two different operational measures of affective polarization: Wagner's (2021) distance from the most liked party, based on the whole electorate, and Reiljan's (2020) Affective Polarization Index (API), based on reported partisans only. On the one hand, we concur with existing studies that affective polarization among partisans follows idiosyncratic country-specific patterns. On the other hand, our estimates of affective polarization among the electorate point to a less equivocal finding, namely, that increasing affective polarization is, with some minor exceptions, a genuinely American story.

## Operationalization Strategies

Existing studies have relied on various techniques to measure affective polarization (Iyengar et al. 2019, pp. 131–34). When it comes to comparative research, however, scholars have customarily resorted to survey-based feeling thermometers. Despite their limitations, feeling thermometers *“*have been asked for long periods of time, allowing researchers to document changes over time” (p. 134).

Measuring affective polarization in the United States is simplified by the dichotomous structure of party competition, opposing the thermometer evaluation of the in-party to that of a single out-party competitor. While the operationalization of the in-party remains equally straightforward in multiparty systems, their complexity renders more intricate the definition of the out-party.

The Affective Polarization Index (API) for multiparty systems was developed by Reiljan (2020). The pillars of this measure are the inclusion of both in-party and out-party evaluations of supporters of all relevant parties (i.e., self-declared partisans) as well as the consideration of the respective parties’ vote share.

An alternative measure has been developed by Wagner (2021), who agrees that the electoral size of parties matters for levels of affective polarization because party systems have different cross-sectional configurations which vary over time. However, Wagner challenges the conceptualization of partisanship as a social identity underlying Reiljan’s measure—namely, that partisan feelings are mutually exclusive and hence only one party can be considered the in-group. As a matter of fact, “many citizens feel positive towards two or more parties, while disliking others” (Wagner 2021, p. 3). For this reason, the author follows Ward and Tavits (2019) and relies on the spread of positive and negative affect among respondents, independently of the strength of their party closeness.^1^

Our empirical analysis relies on both discussed measures to illuminate on the diverging longitudinal trends in affective polarization among *partisans* and among *the electorate*, respectively.

## Data and Measures

Our analyses are based on a novel collection of national election study datasets featuring feeling thermometer questions, as they are necessary to calculate our affective polarization scores for a given country/election year. Data for the fourteen West European democracies included in this study come from the “West European Voter” project (Garzia, Ferreira da Silva, and De Angelis 2022), while data for Australia, Canada, New Zealand, and the United States come from the respective national election study programs.^2^

To maximize longitudinal trends, we resorted to party-leader thermometers whenever party thermometers were not available (N = 26). This, most notably, allows us to track trends for countries like Italy or Spain for which party thermometers have rarely been included in national election studies.^3^ To provide comparable estimates across countries and elections, all thermometer scores have been rescaled on a 0–10 scale. Original question wording and answer scales are presented in Supplementary Materialtable S4.

**Figure 1.**
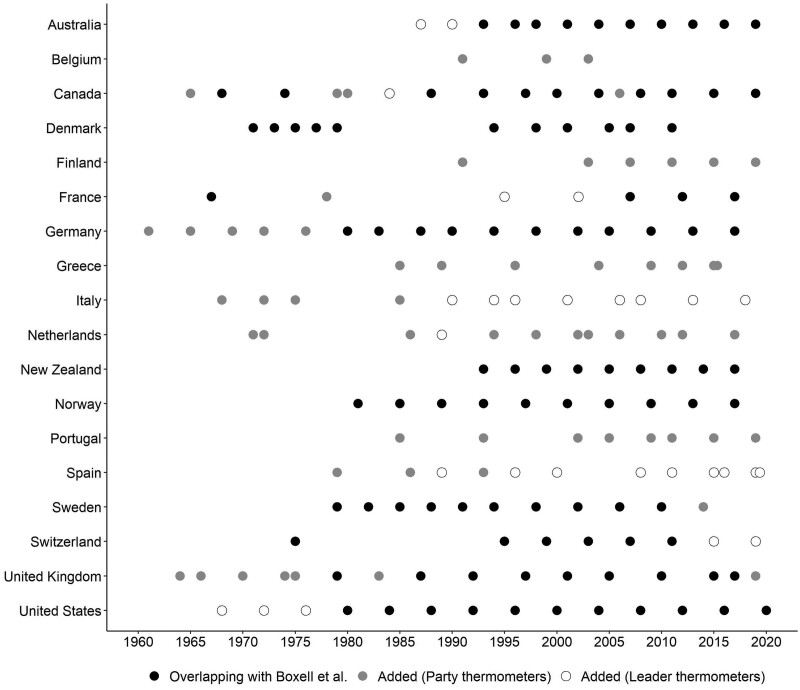
National election study datasets included in the analysis.

Through this dataset, we are able to expand upon Boxell, Gentzkow, and Shapiro’s (2021) most encompassing comparative longitudinal account of affective polarization to date in terms of both geographical scope (seven added countries: Belgium, Finland, Greece, Italy, Netherlands, Portugal, Spain) and time span (twenty-seven data points added to the countries already featured in Boxell et al.’s study). This lends a total of 191 election studies from eighteen democracies. Figure 1 presents all featured election studies, detailing the instances in which each of them overlaps or rather adds to Boxell, Gentzkow, and Shapiro’s (2021) study.

To calculate affective polarization among partisans, we rely on Reiljan’s (2020) formula, whereby in a party system with N relevant parties, the level of AP is: whereby for each individual respondent (*i*), *like* signifies the attitude toward the party, corresponding to the previously described thermometer ratings; *n* denotes the in-party; and *m* refers to the out-party. These party AP scores are weighted with the vote shares of the respective party (*v*) and the scores are summed up to get the weighted average which represents the actual API.


API=∑n=1N ∑m=1m≠nN likein-likeim×vm1-vn×vn

To calculate affective polarization among the electorate, we rely instead on Wagner’s (2021) weighted mean distance from the most liked party. Among the several measures developed by the author, we decided to rely on the mean distance, as it can be directly compared with Reiljan’s measure in terms of metric.^4^ According to this operationalization, the in-party value corresponds to the score of the most liked party on the feeling thermometer, whereas, for a respondent who rated *n* political parties on the feeling thermometer, the out-party variable is: where *p* denotes each non-voted party and *i* is the individual respondent, *v* corresponds to the party vote share, and *like* is the party score on the feeling thermometer. The weighted ratings of each party ($v_{p}{\mathrm{like}}_{ip}$) are averaged across the total number of non-voted parties. Based on this formula, both the in-party affect and out-party affect variables vary from 0 to 10.


$$\mathrm{Out}-p\mathrm{arty}=\;\frac{\sum_{p=1}^{P}v_{p}{\mathrm{like}}_{ip}}{n_{p}}$$

## Trends in Affective Polarization among Partisans

**Figure 2.**
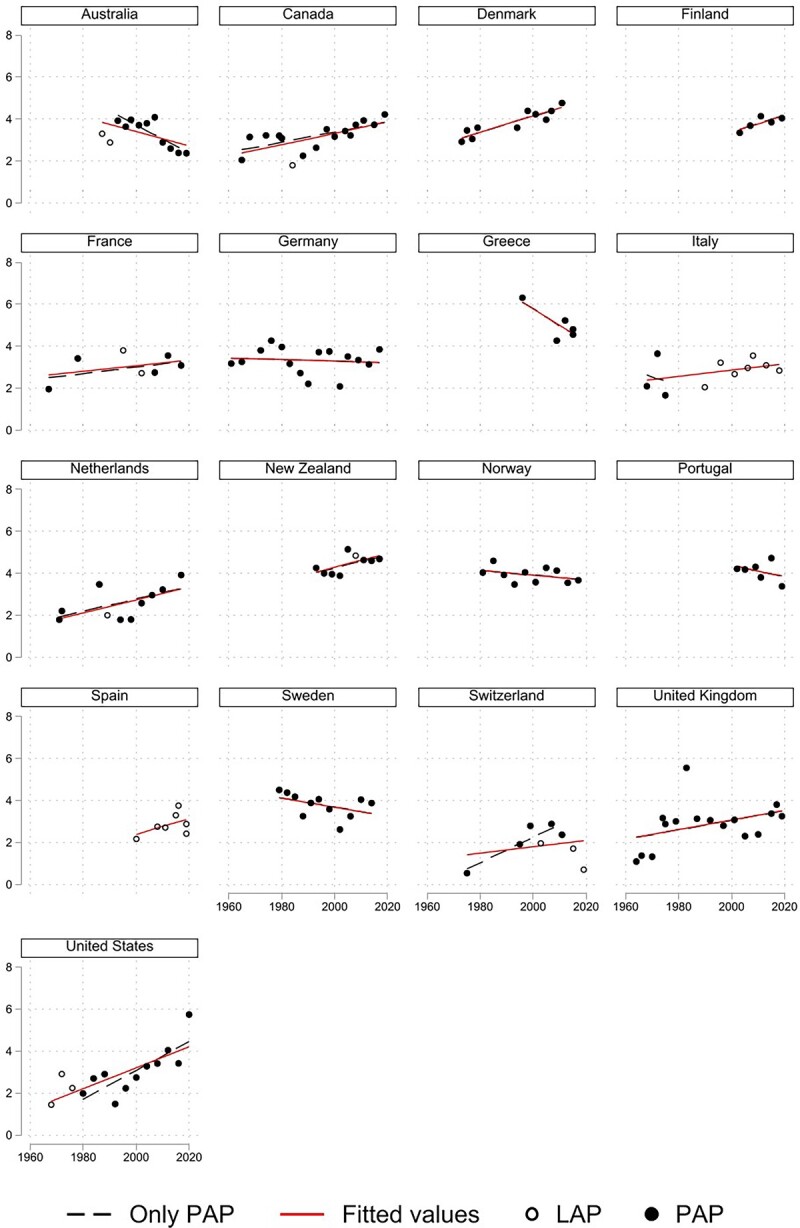
Trends in affective polarization among partisans. Black dots represent AP scores calculated based on party thermometer scores. White dots represent AP scores calculated based on party-leader thermometer scores. The solid line interpolates through OLS regression all country observations, while the dashed line interpolates only the observations based on party thermometer scores (black dots).

Figure 2 presents affective polarization trends among party identifiers, based on Reiljan’s (2020) formula. A visual inspection of the data suggests a relatively idiosyncratic cross-national pattern.

The direction of the trend is positive in eleven countries and negative in six countries.^5^ When it comes to the linearity of these trends, the regression fit lines (see table 1) report conventional levels of statistical significance in seven out of the seventeen countries under analysis: six of them have positive slopes (Canada, Denmark, Netherlands, New Zealand, United Kingdom and the United States) and only one is negatively signed (Australia).

**Table 1.** Detailed estimates for country trends in affective polarization.

	Affective polarization among partisans (all studies included)				Affective polarization among partisans (only estimates based on party thermometers)			
	*b*	adj.se	*p*-value	N	*b*	adj.se	*p*-value	N
Australia	–0.034	0.021	0.094	12	–0.067	0.014	0.002	10
Canada	0.027	0.009	0.008	16	0.024	0.008	0.010	14
Denmark	0.038	0.007	0.001	10	0.038	0.007	0.001	10
Finland	0.038	0.033	0.124	5	0.038	0.033	0.124	5
France	0.014	0.033	0.504	7	0.016	0.033	0.437	5
Germany	–0.004	0.009	0.632	15	–0.004	0.009	0.632	15
Greece	–0.081	0.105	0.190	5	–0.081	0.105	0.190	5
Italy	0.015	0.019	0.334	10	–0.040	1.922	0.915	3
Netherlands	0.031	0.016	0.054	10	0.030	0.016	0.062	9
New Zealand	0.033	0.014	0.027	9	0.031	0.012	0.025	8
Norway	–0.012	0.011	0.225	10	–0.012	0.011	0.225	10
Portugal	–0.026	0.080	0.579	6	–0.026	0.080	0.579	6
Spain	0.038	0.057	0.301	7	–	–	–	–
Sweden	–0.022	0.019	0.200	11	–0.022	0.019	0.200	11
Switzerland	0.015	0.086	0.743	8	0.061	0.055	0.117	5
United Kingdom	0.023	0.014	0.085	16	0.023	0.014	0.085	16
United States	0.050	0.019	0.017	14	0.068	0.029	0.028	11

	Affective polarization among the electorate (all studies included)				Affective polarization among the electorate (only estimates based on party thermometers)			
	*b*	adj.se	*p*-value	N	*b*	adj.se	*p*-value	N
Australia	0.000	0.006	0.999	12	0.000	0.011	0.991	10
Belgium	–0.045	0.123	0.252	3	–0.045	0.123	0.252	3
Canada	0.007	0.008	0.379	16	0.005	0.008	0.502	14
Denmark	0.012	0.009	0.162	11	0.012	0.009	0.162	11
Finland	0.033	0.031	0.137	6	0.033	0.031	0.137	6
France	0.012	0.041	0.634	7	0.017	0.045	0.528	5
Germany	0.025	0.012	0.039	16	0.025	0.012	0.039	16
Greece	–0.024	0.035	0.366	8	–0.024	0.035	0.366	8
Italy	0.009	0.012	0.410	12	–0.013	0.211	0.830	4
Netherlands	–0.001	0.015	0.918	12	–0.002	0.016	0.845	11
New Zealand	0.024	0.019	0.145	9	0.023	0.020	0.159	8
Norway	–0.007	0.007	0.222	10	–0.007	0.007	0.222	10
Portugal	0.025	0.081	0.630	8	0.025	0.081	0.630	8
Spain	–0.004	0.010	0.612	12	0.052	0.141	0.252	3
Sweden	–0.098	0.023	0.002	11	–0.098	0.023	0.002	11
Switzerland	0.020	0.096	0.698	8	0.022	0.161	0.750	5
United Kingdom	0.006	0.020	0.749	16	0.006	0.020	0.749	16
United States	0.029	0.015	0.055	14	0.054	0.014	0.004	11

*Note:* The *b* coefficients come from unstandardized bivariate linear regressions with affective polarization as the dependent variable and survey year as the independent variable. Adjusted standard errors and *p*-values are computed following Imbens and Kolesar (2016).

These results largely corroborate those stemming from Boxell, Gentzkow, and Shapiro’s (2021) longitudinal analysis of twelve countries (see Supplementary Materialtable S2 for a comparison of the country estimates stemming from overlapping election studies). Most notably, we confirm the notion that affective polarization has indeed grown strongest in the United States. The direction of the trends from figure 2 converges with Boxell, Gentzkow, and Shapiro’s (2021) results in all countries but the United Kingdom (see Supplementary Materialfigures S1–S3 for a comparison of fit lines), for which the inclusion of three additional elections with very low polarization levels at the beginning of the time series skews the fit line in an upward direction. We are unable to compare the trends for Germany for two reasons. First, we resort to national election study data while Boxell, Gentzkow, and Shapiro (2021) rely on Politbarometer yearly opinion surveys. Second, they restrict the focus to West German respondents throughout the time series, whereas we also include respondents from East Germany from 1990 onward.^6^

## Trends in Affective Polarization among the Electorate

**Figure 3.**
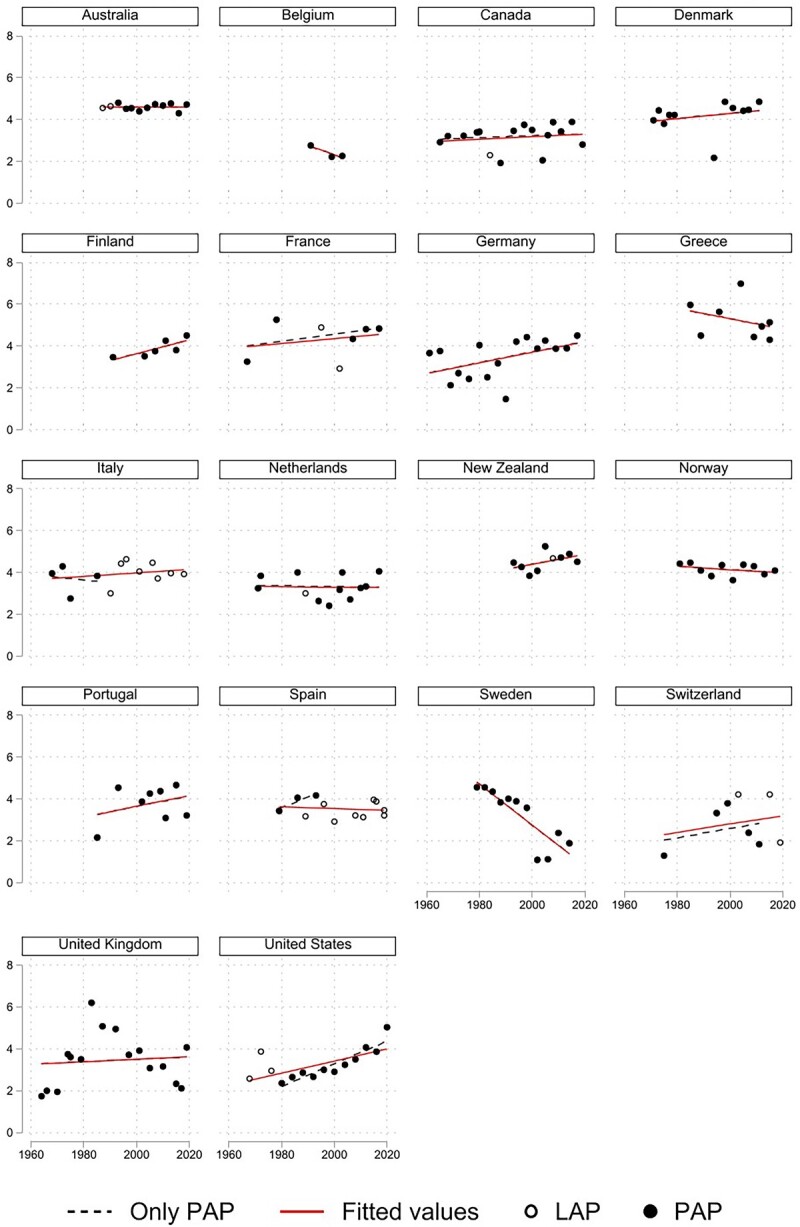
Trends in affective polarization among the electorate. See figure 2.

We now move into less-charted territory, as we attempt measuring affective polarization scores *among the electorate* in comparative/longitudinal perspective. We do so by relying on Wagner’s (2021) weighted mean distance from the most liked party measure. Unlike Reiljan’s (2020) API, this measure does not exclude independents from the calculation, and is hence based on the full sample. The country trends are presented in figure 3.

A visual inspection of the data unfolds little indication of a cross-national trend.

Yet, when it comes to the statistical significance of the country-specific trends, the picture gets clearer (see table 1). Affective polarization increases most noticeably in the United States and Germany, while decreasing markedly in Sweden. In no other country do regression estimates fall within conventional levels of statistical significance.

## Conclusions

The main take-home point of this analysis is that conceptualization matters to understand affective polarization trends. The terms “affective polarization” (Iyengar et al. 2019; Wagner 2021), “partisan polarization” (Stoker and Jennings 2008; Levendusky and Malhotra 2016), or “partisan affective polarization” (Robison and Moskowitz 2019; Stoetzer et al. 2022) have often been used interchangeably in the literature. Likewise, measures of affective polarization are frequently employed without much reflection on the implications of their inherent conceptual and operationalization choices. Does this concept refer to the whole electorate? Or is polarization restricted to those who report a partisan identity?

Our results exemplify the utmost significance of this conceptual and methodological ambiguity for mapping affective polarization trends across the world. While expanding the time span of previous analyses, and most notably Boxell, Gentzkow, and Shapiro (2021), our reassessment of affective polarization *among partisans* lends to a similar conclusion, namely that an intensifying trend is observable in some countries but it is, by no means, generalizable to all Western democracies. However, when it comes to the longitudinal assessment of affective polarization *among the electorate*, the conclusion is less ambiguous. US citizens have indeed become more affectively polarized over time.

Regarding the discrepancy in the trends between polarization among partisans and polarization among the electorate, we can speculate that a long-term process of partisan dealignment may have led to a progressively more radical backbone of partisans in at least some countries.^7^ In turn, such shrinking proportion of partisans could be credited for having increased the levels of affective polarization among this subgroup without having a significant effect on the whole electorate. If this were the case, polarization trends in the United States could be ascribed to the country’s differential effects of partisan sorting on social and issue polarization (Mason 2015, 2018; Harteveld 2021).^8^

Our findings invite a deeper reflection on the bases upon which affective polarization is conceptualized and calculated, while at the same time scrutinizing the commonsensical notion that it has been soaring across the board.

## Supplementary Material

nfad004_Supplementary_Data

## Data Availability

Replication data and documentation are available at https://dataverse.harvard.edu/dataset.xhtml?persistentId=doi:10.7910/DVN/3D97LV.

## Footnotes

1 While excluding nonpartisans from the calculation may not be a problem for cross-sectional studies, this could represent a downside for longitudinal analyses. The widespread process of partisan dealignment documented across Western democracies (Garzia, Ferreira da Silva, and De Angelis 2021) implies that the values of affective polarization over time would be calculated on a shrinking proportion of survey respondents—thus making within-country comparisons problematic.

2 Data for non-European democracies have been retrieved from the respective NES websites: Australia (www.australianelectionstudy.org), Canada (www.ces-eec.ca), New Zealand (www.nzes.org), and the United States (www.electionstudies.org).

3 This approach grounds on the tight correlation between party and leader thermometers, well documented in the political behavior literature, and the high correlation between the party-based and leader-based measures of affective polarization in the election studies featuring both batteries (*r* = 0.83 for affective polarization among partisans; *r* = 0.77 for affective polarization among the electorate; see Supplementary Materialtable S1). As a robustness check, we have re-estimated all country trends excluding the years for which affective polarization scores have been calculated based on party-leader thermometer scores—with no meaningful variation in the results (see table 1).

4 We also tested Wagner’s spread-of-scores measure, which lends a correlation above 0.9 with the weighted mean distance measure and, most importantly, identical results.

5 Belgium is excluded from this part of the analysis due to lack of party identification questions necessary to estimate Reiljan’s API.

6 It should be noted that our estimates and Boxell, Gentzkow, and Shapiro (2021) result in divergent longitudinal trends. While they find evidence of depolarization among the German electorate, our results point to a significant increase in polarization. This is because our longitudinal data capture sixteen years prior to the first data point featured in Boxell, Gentzkow, and Shapiro (2021). Affective polarization in these early elections is distinctively low, thus pulling down the fit line at the beginning of the time trend. If we were to look only at the period analyzed by Boxell et al.—the eleven data points from 1980 to 2017—we would find no significant polarization trend for Germany. Overall, this finding highlights the added value of the longer time series offered by our data. Please refer to the “On the divergence of Germany’s estimates between our data and Boxell, Gentzkow, and Shapiro (2021)” Supplementary Material section for a more detailed comparison.

7 For example, a previous study has asserted that “polarization is a phenomenon concentrated in the one-third of Americans who consider themselves strong partisans” (Klar, Krupnikov, and Ryan 2018, p. 379).

8 A systematic comparison between the two measures (Supplementary Materialfigure S4 and table S3) reveals that, despite being more stable over time, levels of polarization among the electorate generally tend to be higher than among partisans. We are inclined to interpret this counterintuitive finding based on the way in which the in-party is operationalized in Reiljan’s (2020) and Wagner’s (2021) formulas, respectively. While the former assigns to the in-party the thermometer score ascribed to the party the respondent identifies with (and hence, not necessarily the highest thermometer score), the latter assigns the highest thermometer score to the in-party. By inflating the in-party side of the AP equation, its overall value gets inflated as a result. To validate this reasoning, we recalculated Wagner’s AP scores for separate groups of voters (i.e., independents, leaners, partisans) and find that the mean AP score increases linearly as partisanship gets stronger. In other words, partisans are *more* polarized than independents, in spite of what the cross-measure comparison would seem to suggest.
