# Supplementary material for: Affective Polarization in Comparative and Longitudinal Perspective
Source: Public Opin Q. 2023 Feb 24;87(1):219–31. doi: 10.1093/poq/nfad004 (PMC10127533; doi:10.1093/poq/nfad004)
Supplement: nfad004_Supplementary_Data [file nfad004_supplementary_data.pdf]

Supplementary material

Affective Polarization in Comparative and Longitudinal Perspective

September 2022

Diego Garzia

Frederico Ferreira da Silva

Simon Maye

Table S1. Correlation analyses of affective polarization scores based on party (PAP) and leader (LAP) thermometers, among partisans (panel above) and the electorate (panel below)

Country	N	*r*	PAP Partisans	LAP Partisans	PAP/LAP Ratio	t-test
United States	11	.98	3.078	3.452	0.892	.020
Portugal	6	.99	4.110	3.611	1.138	.000
Canada	14	.86	3.318	2.896	1.146	.000
Denmark	7	.95	4.033	3.440	1.172	.001
New Zealand	8	.70	4.398	3.717	1.183	.002
Greece	5	.90	5.033	4.200	1.198	.007
Germany	15	.64	3.326	2.774	1.199	.002
Sweden	10	.82	3.711	3.060	1.213	.000
Australia	10	.94	3.334	2.741	1.216	.000
Norway	10	.86	3.928	3.098	1.268	.000
United Kingdom	16	.65	2.851	2.242	1.272	.009
Netherlands	7	.98	2.824	2.217	1.274	.000
France	4	.95	2.829	2.181	1.297	.216
Finland	5	.79	3.815	2.937	1.299	.001
Switzerland	4	.98	2.487	1.462	1.701	.001
**All countries**	**132**	**.83**	**3.477**	**2.926**	**1.188**	**.000**

Country	N	*r*	PAP Electorate	LAP Electorate	PAP/LAP Ratio	t-test
Switzerland	4	–.71	2.836	3.912	0.725	.217
United States	11	.94	3.282	4.148	0.791	.000
United Kingdom	16	.80	3.445	3.496	0.985	.791
Canada	14	.73	3.227	3.207	1.006	.862
Italy	1	–	3.837	3.810	1.007	–
Sweden	10	.92	3.074	3.049	1.008	.904
Denmark	8	.97	4.214	4.167	1.011	.569
New Zealand	8	.75	4.505	4.432	1.016	.544
Portugal	8	.97	3.768	3.693	1.020	.370
Greece	7	.80	4.984	4.799	1.039	.393
Australia	10	.78	4.603	4.412	1.043	.058
Netherlands	9	.85	3.288	3.153	1.043	.256
Norway	10	.88	4.149	3.896	1.065	.000
Germany	15	.83	3.519	3.298	1.067	.116
Spain	3	–.20	3.893	3.490	1.115	.393
France	4	.98	4.307	3.756	1.147	.447
Finland	5	.90	3.964	3.313	1.196	.043
**All countries**	**143**	**.77**	**3.746**	**3.716**	**1.008**	**.559**

Note: All t-tests are two-tailed.

Table S2. Correlations between our estimates of affective polarization among partisans and Boxell et al.'s (2021) Figure 1 estimates

	**AP among partisans vs. Boxell et al.'s Figure 1**	**Alternative AP among partisans vs. Boxell et al.'s Figure 1**	**N**
Australia	*.30*	.83	10
Canada	.79	.79	11
Denmark	.84	.75	10
France	.82	.97	4
New Zealand	.92	.95	8
Norway	.76	.94	10
Sweden	.87	.95	10
Switzerland	.97	.94	5
UK (all observations)	*.62*	*.56*	9
UK (1987 and 1992 excluded)	.87	.95	7
United States	.94	.97	11
**All countries**	**.59**	**.78**	**88**
**All countries** (UK 1987 and 1992 excluded)	**.62**	**.81**	**86**

Note: Entries in first column are Pearson's *r* correlation coefficients between our AP estimates among partisans (from Figure 2) and those from Boxell et al's (2021) Figure 1. Entries in the second column are Pearson's *r* correlation coefficients between our alternative AP estimates among partisans (calculated using the proportion of self-declared partisans for each party instead of its electoral size) and those from Boxell et al.'s (2021) Figure 1.

Figure S1. Comparing time trends: Figure 1 vs. Boxell et al.'s (2021) Figure 1

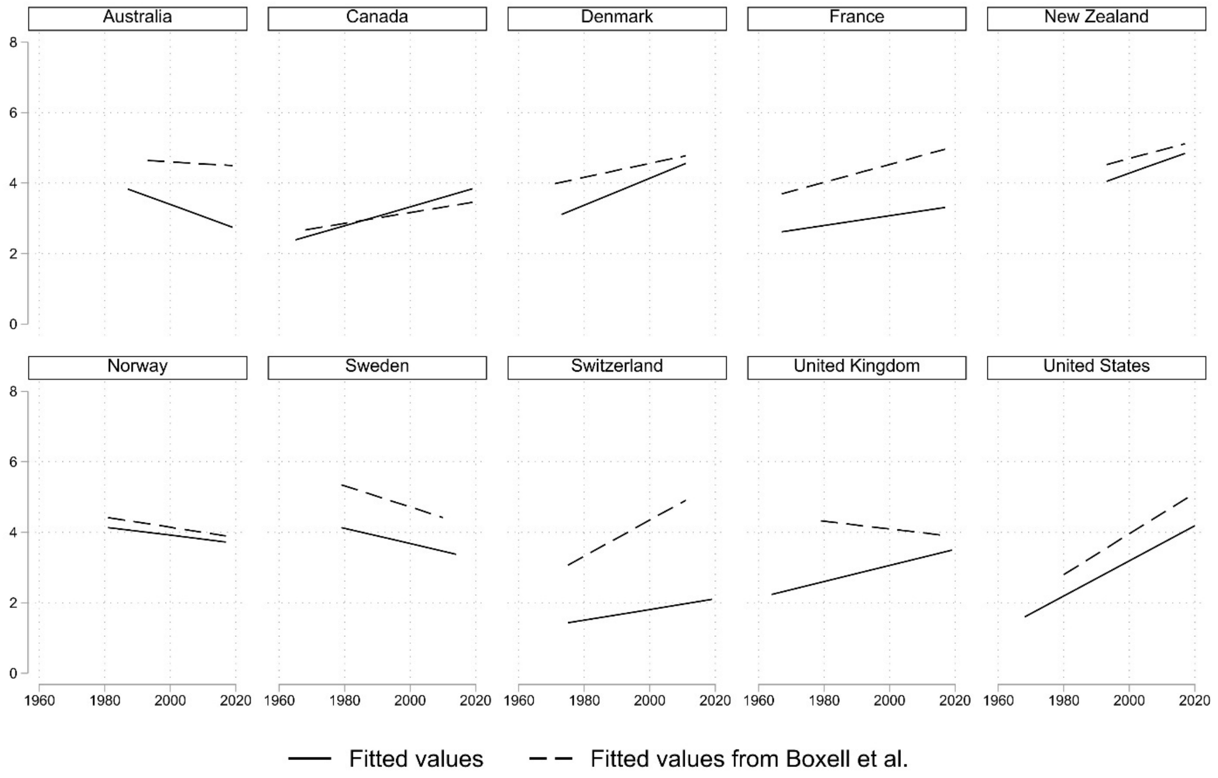

Figure S2. Comparing time trends: Figure 1 vs. Boxell et al.'s (2021) Figure 1 – only overlapping studies included

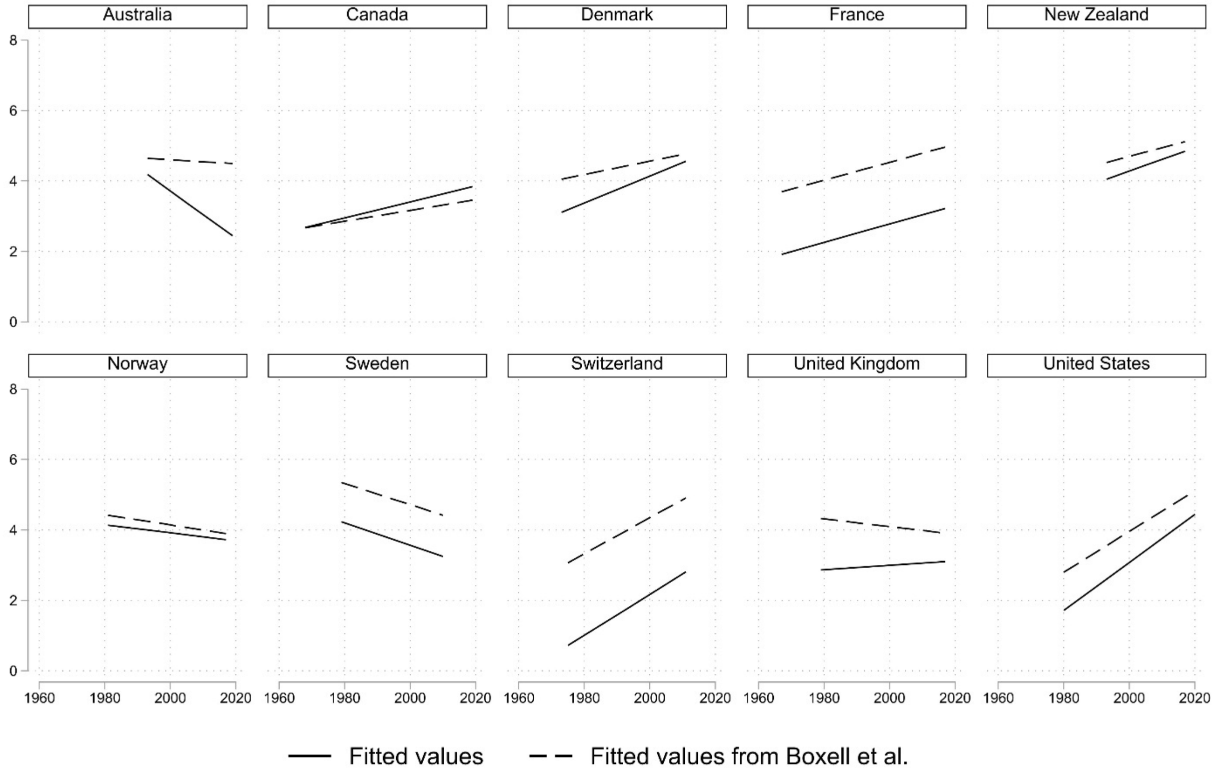

Figure S3. Comparing time trends: Alternative AP measure vs. Boxell et al.'s (2021) Figure 1 – only overlapping studies included

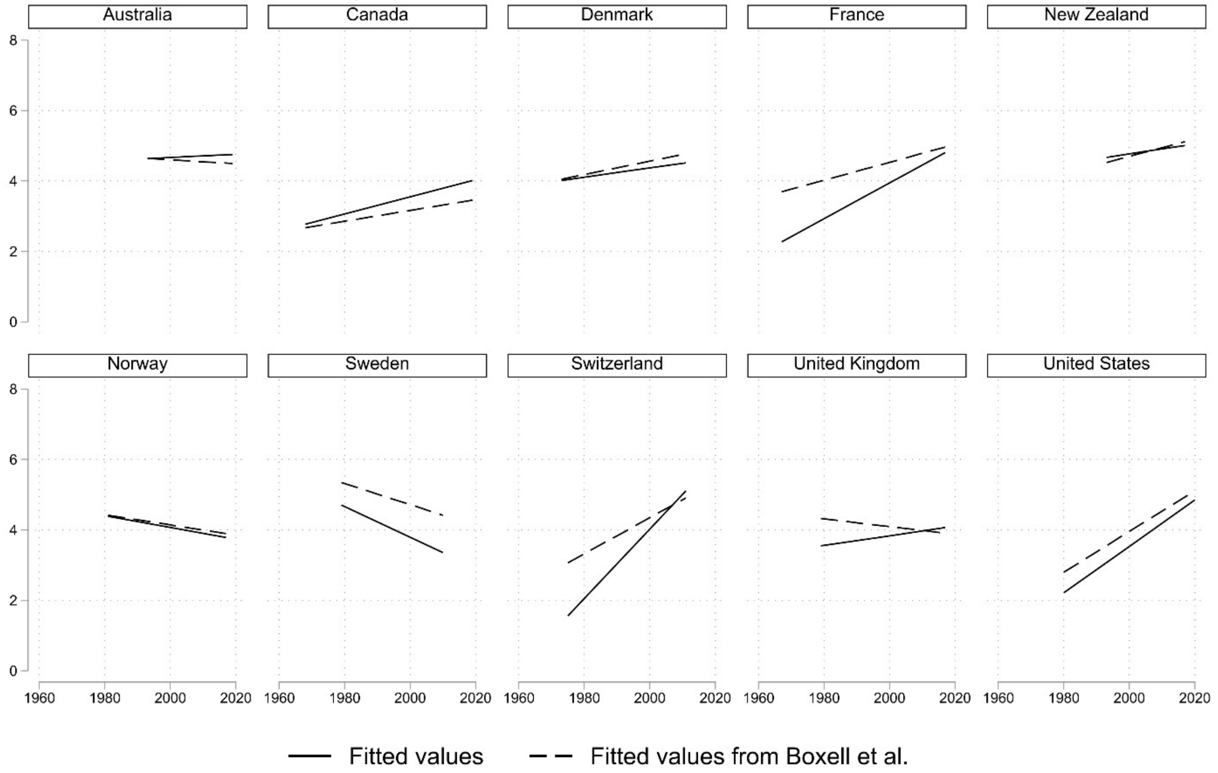

Figure S4. Affective polarization among partisans and the electorate: Scatterplot

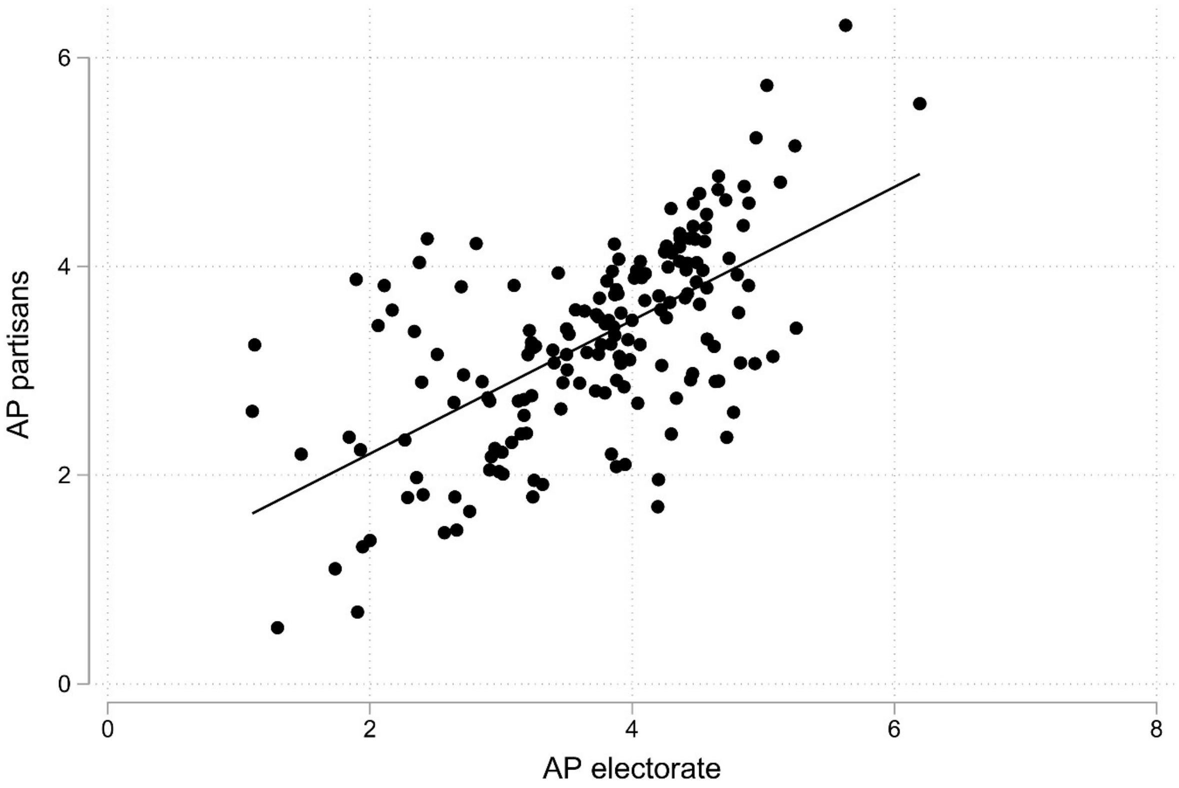

Table S3. Correlation between AP measures and respective mean values, by country

**Country**	**N**	***r***	**$AP_{partisans}$**	**$AP_{electorate}$**	**AP_p/AP_e ratio**	**t-test**
Switzerland	8	.43	1.853	2.868	0.646	.036
France	7	.79	3.035	4.326	0.702	.001
Australia	12	.07	3.295	4.603	0.716	.000
Italy	10	.73	2.781	3.876	0.717	.000
Netherlands	10	.64	2.577	3.237	0.796	.007
United Kingdom	16	.72	2.851	3.445	0.828	.017
Spain	7	.92	2.857	3.401	0.840	.001
United States	14	.91	2.891	3.251	0.889	.027
Denmark	10	.40	3.832	4.199	0.913	.169
Germany	15	.31	3.326	3.519	0.945	.423
Norway	10	.87	3.928	4.149	0.947	.003
Finland	5	.88	3.815	3.964	0.962	.157
New Zealand	9	.90	4.450	4.522	0.984	.292
Canada	16	.49	3.143	3.149	0.998	.973
Greece	5	.89	5.033	4.888	1.030	.462
Portugal	6	.90	4.110	3.910	1.051	.160
Sweden	11	.71	3.783	3.210	1.179	.091
**All countries**	**172**	**.63**	**3.290**	**3.701**	0.889	**.000**

Note: All t-tests are two-tailed.

Table S4. Original question wording and answer scales for party/leader thermometers

Country	Year	Question wording	Scale
Australia	1987*<br>1990*	We would like to know your feelings about the party leaders. Please show how you feel about them by circling a number from 0 to 10. 10 is the highest rating, for people you feel very favourable about, and 0 is the lowest rating, for people you feel very unfavourable about. If you are neutral about a particular person or dont know much about them, you should give them a rating of 5. How do you feel about:	0-10
Australia	1993	Finally in this section, we would like to know your feelings about the political parties. Please show how you feel about them by circling a number from 0 to 10. 10 is the highest rating, if you feel very favourable about a party, and 0 is the lowest rating, for parties you feel very unfavourable about. If you are neutral about a particular party or don't know much about them, you should give them a rating of 5.	0-10
Australia	1996<br>1998<br>2001<br>2004<br>2007<br>2010<br>2013<br>2016<br>2019	Finally in this section, we would like to know what you think about each of our political parties. Please rate each party on a scale from 0 to 10, where 0 means you strongly dislike that party and 10 means that you strongly like that party. If you are neutral about a particular party or don't know much about them, you should give them a rating of 5.	0-10
Belgium	1991	Best to worst party (RANKING)	1-9
Belgium	1999	There are a lot of political parties in our country. We like to know how you like them. A low score means very unsympathetic, a high score means very sympathetic. If you don't know a political party, just say it.	0-10
Belgium	2003	I'd like to know what you think about each of our political parties. After I read the name of a political party, please rate it on a scale from 0 to 10, where 0 means you strongly dislike that party and 10 means that you strongly like that party. If I come to a party you haven't heard of or you feel you do not know enough about, just say so.	0-10
Canada	1965	Now i have a different kind of question for you. I'm going to show you some word pairs. Each pair is separated by seven boxes like this. If you think that the particular political party is bad, you would put a check mark in the box on the left end of the scale. If you feel it is good, you would check the box on the right end of the scale. Or you might rate it somewhere between these two extremes.	1-7
Canada	1968<br>1974<br>1979	You'll see here a drawing of a thermometer. It's been called a feeling thermometer because it helps measure one's feelings towards various things. Here's how it works. If you don't particularly like or dislike the person, group or activity we are asking about, place them at the 50 degree mark. If your feelings	0-100

		are very warm then you would give a score between 50 and 100, the warmer your feelings, the higher the score. On the other hand, if you do not like the person, group or activity very much, you would place them somewhere between 0 and 50. The cooler your feelings, the closer the number will be to 0. If you don't know too much about one of the items mentioned, just say so, and we'll go on to the next one ... How would you rate the party, taken as a whole?	
Canada	1980	Now we would like you to think about the feeling thermometer which we used in our 1979 study and which we sent to you in the mail. The thermometer is graded from 00 to 100. If you don't have any particular feeling about the things we are asking about, place them at the 50 degree mark. If you feelings are very warm toward a particular thing, you would give a score between 50 and 100, the warmer your feelings, the higher the score. On the other hand, if your feelings are relatively cool toward something, you would place them between 0 and 50. The cooler your feelings, the closer the score will be to zero. If you don't know too much about any of the items mentioned, just say so and we will go on to the next one. There are many aspects of political parties which strike canadians in different ways. We would like to get your feelings toward some of these aspects of our parties. We are interested to see how you liked the leaders, the party's candidate in your riding in the last election, and the party as a whole. We will use the feeling thermometer again for these questions.	0-100
Canada	1984*	It is called a feeling thermometer because it helps us to measure feelings toward various groups of people. Here is how it works. Scores between 50 degrees and 100 degrees mean that you feel favourable and warm toward a group of people -- the higher the score, the warmer and more favourable your feelings. Scores between 0 degrees and 50 degrees mean that you don't feel too favourable and are cool toward a group of people -- the lower the score, the cooler and less favourable your feelings. If you don't have any feelings at all toward a group of people, just say so and we'll go on the the next one.	0-100
Canada	1988<br>1993	Now let's talk about your feelings towards the political parties, their leaders and their candidates. I'll read a name and ask you to rate a person or a party on a thermometer that runs from 0 to 100 degrees. Ratings between 50 and 100 degrees mean that you feel favourable toward that person. Ratings between 0 and 50 degrees mean that you feel unfavourable toward that person. You may use any number from 0 to 100 to tell me how you feel. How would you rate [PARTY]?	0-100
Canada	1997	[Now we're going to ask you how you feel about the party leaders using a scale from 0 to 100. 0 means you really DISLIKE the leader and 100 means you really LIKE the leader. You can use any number from 0 to 100.} Now we're going to ask you how you feel about each political party on the same scale. The scale runs from 0 to 100, where 0 means an extremely bad rating and 100 means an extremely good rating	0-100

Canada	2000	And now, how do you feel about the political parties. Use a scale from ZERO to ONE HUNDRED. Zero means you REALLY DISLIKE the party and one hundred means you REALLY LIKE the party.	0-100
Canada	2004	Using the scale below, please rate each party:	0-100
Canada	2006	And now, how do you feel about the political parties. Use a scale from ZERO to ONE HUNDRED. Zero means you REALLY DISLIKE the party and one hundred means you REALLY LIKE the party.	0-100
Canada	2008	Using the scale below, please rate each party:	0-100
Canada	2011<br>2015	And now, how do you feel about the political parties. Use a scale from ZERO to ONE HUNDRED. Zero means you REALLY DISLIKE the party and one hundred means you REALLY LIKE the party.	0-100
Canada	2019	I'd like to know what you think about each of our political parties. After I read the name of a political party, please rate it on a scale from 0 to 10, where 0 means you strongly dislike that party and 10 means that you strongly like that party. If I come to a party you haven't heard of or you feel you do not know enough about, just say so.	0-10
Denmark	1971<br>1973<br>1975<br>1977<br>1979	I have a card with a kind of thermometer called a 'sympathy thermometer', and we will ask you to give the parties' temperatures according to how much you like them. Give plus temperatures to all of the parties you like – the more you like a party, the higher the temperature. The parties you don't like get minus temperatures. If you neither like nor dislike a party, give it a 0.	0-10
	1994	Here are some questions about how much you like the parties, party leaders, and the policy which the parties have pursued. Even though you may view a party, its leader and policy as a whole, we ask you to try and answer each question. Beginning with the leaders, here is a card with a scale running from 0 to 10. The more you like the person, the higher mark you give. If you neither like nor dislike a person, you should give 5.	0-10
Denmark	1998<br>2001<br>2005<br>2007<br>2011	Now I would like to hear what you think of the political parties. After I have mentioned the party, I want you to place it on this scale from 0 to 10, where 0 means you that dislike the party were much and 10 means that you like it were much. If I mention a party that you don't know or don't feel you know enough about, just say so.	0-10
Finland	1991	How positive or negative you feel towards the following groups/parties on a scale from -50 (very negative) to +50 (very positive)	-50<br>+50
Finland	2003<br>2007<br>2011	Opinion on political parties. Rate the following parties on a scale from 0 (strongly dislike) to 10 (strongly like). Say if you haven't heard of the party or feel you do not know enough about it	0-10
Finland	2015<br>2019	What do you think about the following political parties on a scale 0-10, where 0 means 'strongly dislike' and 10 means 'strongly like'?	0-10

France	1967	There are groups and individuals who influence government and public opinion., We would like to know your feelings in repsect to them. Would you put a figure between 0 and 100 by these persons or groups according to the feelings which you have for them.	0-100
France	1978	Can you rank the following political parties in terms of how sympathetic you are to them, with the first being the most sympathetic and the last the most unsympathetic to you? (RANKING)	1-7
France	1995*	I am going to read you the names of the candidates for the presidential election. For each one I would like you to tell me what is your degree of degree of sympathy for each one, using this thermometer which varies from 0 to 100°. 0° is the maximum antipathy, 100° is the maximum sympathy, 50° is neither sympathy nor antipathy.	0-100
France	2002*	Here is a list of personalities. For each of them, tell me your degree of sympathy towards him, using this thermometer which varies from 1 to 10 (1 corresponds to a strong antipathy and 10 to a strong sympathy)?	1-10
France	2007<br>2012<br>2017	Could you give each of the following parties a score from 0 to 10, where 0 means you don't like that party at all and 10 means you like it very much. If you don't know any of these parties, please let me know.	0-10
Germany<br><br><br><br><br><br><br><br><br>Germany	1961<br>1965<br>1969<br>1972<br>1976<br>1980<br>1983<br>1987<br>1990<br>1994<br>1998	DATA RETRIEVED FROM THE "EUROPEAN VOTER" DATASET: Please provide a measure of the respondent's overall sympathy/likeing for each party. We would anticipate that this would normally be the product of a theremometer' score, but in the absence of such a score please supply the most similar alternative (Thomassen, 2005)	1-11
Germany	2002<br>2005<br>2009<br>2013<br>2017	What do you think, in general, about the political parties? Please tell me by using this scale. +5 means that you think a great deal of the party, -5 means that you don't think much of it at all. Using the values in between you can express your opinion more precisely.	-5 +5
Greece	1985<br>1989	Feelings about (PARTY)	1-10
Greece	1996<br>2004	Feelings towards some persons and social organization on a scale from 0-10. If your feel very favorable towards this person, you can give hom the highest score of 10; if you feel hostile towards ths persone you can give him a 0. If you feel absolutely neutral towards this person, you can give him a 5.	0-10
Greece	2009<br>2012	I'd like to know what you think about each of our political parties. After I read the name of a political party, please rate it on a scale from 0 to 10, where 0 means you strongly dislike that party and 10 means that you strongly like that party. If I come to a party you	0-10

		haven't heard of or you feel you do not know enough about, just say so.	
Greece	2015°<br>2015b	I would like you to tell me what you think of each of the political parties of in our country. To tell me what you think, use a scale from 0 to 10, where 0 means "you don't like this particular party at all". party" and 10 means that you "like it very much".	0-10
Italy	1968<br>1972	There are persons, groups and organization who influence the government and public opinion. We would like to know your feelings toward them. Pleaser, therefore, give a score of from 0 to 100 according to the feelings you gave fir them. (100 means you approve higly, 50 means you are neither approving or disapprobing ot not familiar with him/it, 0 means you do not like him/it at all.	0-100
Italy	1975	If you are neither favorable nor unfavorable towards a given (political) group, please place the feeling thermometer around 50 degrees. If you are favorable, place it between 50 and 100 degrees, the closer to 100 the more favorable you are. If you are not very favorable, place between 50 and 0 degrees, the closer to 0 the more unfavorable you are.	0-100
Italy	1985	I would like to know what do you think about certain groups, institutions, or countries. Using a scale from 1 to 10, please say how much sympathy you have for each for them, taking into account that 1 means no sympathy at all and 10 much sympathy	1-10
Italy	1990*<br>1994*<br>1996*<br>2001*<br>2006*	I shall now read you a list of national politicians. For each of them, tell me whether you have ever heard of them and, if so, give them a score from 1 to 10 according to your opinion on them: 1 means a totally negative judgement and 10 means a totally positive judgement	1-10
Italy	2008*<br>2013*<br>2018*	I shall now read you a list of national politicians. For each of them, tell me whether you have ever heard of them and, if so, give them a score from 0 to 10 according to your opinion on them: 0 means a totally negative judgement and 10 means a totally positive judgement	0-10
Netherlands	1971<br>1972<br>1986<br>1989*<br>1994<br>1998	DATA RETRIEVED FROM THE "EUROPEAN VOTER" DATASET: Please provide a measure of the respondent's overall sympathy/likeing for each party. We would anticipate that this would normally be the product of a theremometer' score, but in the absence of such a score please supply the most similar alternative. (Thomassen 2005)	0-100
Netherlands	2002<br>2003	There are many political parties in our country. I would like to know from you again how sympathetic you find these parties. You can give each party a score between 0 and 100. The more sympathetic you find a party, the higher the score you give. A score of 50 means that you find a party neither sympathetic nor unsympathetic. If you don't know a party, please feel free to say so.	0-100
Netherlands	2006<br>2010<br>2012	How sympathetic do you find the following political parties? You can give each party a score between 0 and 10. 0 means that you find this party not sympathetic and 10 means that you find this	0-10

	2017	party very sympathetic. If you don't know a political party, please feel free to say so. What number would you assign the following parties?	
New Zealand	1993	Regardless of what their chances were in winning your particular electorate, or even winning any seats at all, how do you feel about these political parties?	1-5
New Zealand	1996<br>1999<br>2002<br>2005	We would like to know what you think about each of these political parties. Please rate each party on a scale from 0 to 10, where 0 means you strongly dislike that party and 10 means that you strongly like that party. If you haven't heard about that party or don't know enough about it, please circle '99' under 'don't know'. How do you feel about: [Insert Party Name]	0-10
New Zealand	2008*	Now we would like to know what you think about each of these political leaders. Please rate each party on a scale from 0 to 10, where 0 means you strongly dislike that party leader and 10 means that you strongly like that leader party. If you haven't heard about that party leader or don't know enough about it, please circle '99' under 'don't know'. How do you feel about: [Insert Party Leader Name]	0-10
New Zealand	2011<br>2014<br>2017	We would like to know what you think about each of these political parties. Please rate each party on a scale from 0 to 10, where 0 means you strongly dislike that party and 10 means that you strongly like that party. If you haven't heard about that party or don't know enough about it, please circle '99' under 'don't know'. How do you feel about: [Insert Party Name]	0-10
Norway	1981<br>1985<br>1989<br>1993	We want to know how much or little you like the different parties. On this card is a scale that we call "sympathy thermometer." At 50-degrees-line position the parties that you neither like or dislike. A party that you like to have a location from 50 to 100 degrees. The better you like the party, the higher position. However, if it is a party you do not like, it should be placed between 0 and 50 degrees, with 0 as the expression of at least sympathy.	0-100
Norway	1997<br>2001<br>2005<br>2009<br>2013<br>2017	After I read the name of a political party, please rate it on a scale from 0 to 10, where 0 means you strongly dislike that party and 10 means that you strongly like that party.	0-10
Portugal	1985	I would like to know your opinion about certain groups, institutions and countries. Using a scale from 0 to 10, in which 0 means no sympathy and 10 means much sympathy, please tell me how you feel towards the following groups, institutions and countries, indicating the scale value corresponding to the degree of sympathy you feel for each of them.	0-10
Portugal	1993	I would like you to tell me about your sympathy for each political party. I have here numbers from 1 to 10 in which 10 means you feel much sympathy and 1 means you feel no sympathy at all. Which value would you use to describe the sympathy you have for:	1-10
Portugal	2002		0-10

	2005<br>2009<br>2011<br>2015<br>2019	I'd like to know what you think about each of our political parties. After I read the name of a political party, please rate it on a scale from 0 to 10, where 0 means you strongly dislike that party,10 means that you strongly like that party and 5 that you feel indifferent toward the party. If I come to a party you haven't heard of or you feel you do not know enough about, just say so. The first party is	
Spain	1979	n/a	0-10
Spain	1986	Now I'm going to read a series of parties. Tell me if you feel very close, close, neither close nor distant, distant or very distant from each of them.	1-5
Spain	1989*	I'm going to read out a series of political leaders' names. Please tell me, for each one, whether you know of them and how you value their political performance. Grade them from 0 to 10, where 0 means that you value them "very bad" and 10 means that you value them "very good"	0-10
Spain	1993	Now I'm going to read a series of parties. Tell me if you feel very close, close, neither close nor distant, distant or very distant from each of them.	1-5
Spain	1996*<br>2000*<br>2008*<br>2011*<br>2015*<br>2016*<br>2019a*<br>2019b*	I'm going to read out a series of political leaders' names. Please tell me, for each one, whether you know of them and how you value their political performance. Grade them from 0 to 10, where 0 means that you value them "very bad" and 10 means that you value them "very good"	0-10
Sweden	1979<br>1982<br>1985<br>1988<br>1991<br>1994<br>1998<br>2002<br>2006<br>2010<br>2014	On this card there is a kind of scale. I would like you to use it in order to state how much you like or dislike the parties. If you like a party, use the "plus" figures. The better you like a party the higher the "plus" figure. For parties you dislike, use the "minus" figures. The more you dislike a party, the higher the "minus" figure. The zero point on the scale indicates that you neither like nor dislike a party. Where would you like to place the...?	-5 +5
Switzerland	1975	Here is a scale we call a favorability thermometer. Please give a score between 0 and 100 indicating how much you like the following groups and organizations. 100 means that you like them very much, 0 means that you do not like them at all. If you don't particularly like or dislike them... give a score of 50. What score would you give to [Insert Party Name]?	0-100
Switzerland	1995<br>1999	Now I would like to know what you think of our political parties. When I read the name of a political party to you, please indicate where you place it on a scale from 0 to 10, with 0 meaning "no sympathy at all", and 10 meaning "a lot of sympathy".	0-10

Switzerland	2003*	And can you tell me what sympathy you have for the following political figures on a scale from 0 to 10, where 0 means "no sympathy" and 10 "very strong sympathy"?	0-10
Switzerland	2007	We would now like to know what you think of some of the political parties. Please place the on a scale from 0 to 10. 0 means that you do not like this party at all. 10 means you like this party very much.	0-10
Switzerland	2011	Could you indicate, on a scale of 0 to 10, how much sympathy you feel for the following parties.	0-10
Switzerland<br>Switzerland	2015*<br>2019*	And can you tell me what sympathy you have for the following political figures on a scale from 0 to 10, where 0 means "no sympathy" and 10 "very strong sympathy"?	0-10
UK	1964<br>1966<br>1970<br>1974a<br>1974b	DATA RETRIEVED FROM THE "EUROPEAN VOTER" DATASET: Please provide a measure of the respondent's overall sympathy/likeing for each party. We would anticipate that this would normally be the product of a theremometer' score, but in the absence of such a score please supply the most similar alternative. (Thomassen 2005)	0-10
UK	1979	Let's say that you gave each of the parties a mark out of ten points—a mark according to how much or how little you like it. You can give each party any mark from 0 out of 10 for the least like, to 10 out of 10 for the most liked. What mark out of 10 would you give the [Insert Party Name]?	0-10
UK	1983<br>1987<br>1992	Please choose a phrase from this card to say how you feel about the Party?	1-4<br>1-5<br>1-5
UK	1997<br>2001<br>2005<br>2010<br>2015<br>2017<br>2019	I'm now going to ask a few questions about political parties. On a scale that runs from 0 to 10, where 0 means strongly dislike and 10 means strongly like, how do you feel about the Party?	0-10
United States	1968*<br>1972*<br>1976*	I'd like to get your feelings toward some of our political leaders and other people who are in the news these days. I'll read the name of a person and I'd like you to rate that person using something we call the feeling thermometer. Ratings between 50 degrees and 100 degrees mean that you feel favorable and warm toward the person. Ratings between 0 degrees and 50 degrees mean that you don't feel favorable toward the person and that you don't care too much for that person. You would rate the person at the 50 degree mark if you don't feel particularly warm or cold toward the person. If we come to a person whose name you don't recognize, you don't need to rate that person. Just tell me and we'll move on to the next one.	0-100
United States	1980<br><br>1984<br>1988<br>1992	We'd also like to get your feelings about some groups in American society. When I read the name of a group, we'd like you to rate it with what we call a feeling thermometer. Ratings between 50 degrees-100 degrees mean that you feel favorably and warm toward the group; ratings between 0 and 50 degrees mean that you	0-100

1996<br>2000<br>2004<br>2008<br>2012<br>2016<br>2020	don’t feel favorably towards the group and that you don’t care too much for that group. If you don’t feel particularly warm or cold toward a group you would rate them at 50 degrees. If we come to a group you don’t know much about, just tell me and we’ll move on to the next one.

On the divergence of Germany’s estimates between our data and Boxell et al. (2021)

Boxell et al.’s (2021) findings for Germany show evidence of depolarization while our own suggest instead a polarization trend. Despite using different data sources, both studies rely on nationally representative samples of the German voting population. Therefore, what explains such a stark difference in findings? Below, we offer some explanations for the differences between estimates:

The first, and most important, factor accounting for the divergences in the trends has to do with the difference in the length of the time-series in the two studies. The first data point for Germany in Boxell et al.’s (2021) article starts in 1977, whereas our first data point is from 1961. Therefore, the estimation of our trend adds 16 years, measured through five elections. Affective polarization in these early elections is markedly low: on average 2.93 in the first five elections. These early data points are fundamental for the polarization trend found in our data, since they pull down the fit line at the beginning of the time trend. If we were to look only at the period analyzed by Boxell et al. – the 11 data points from 1980-2017 – we would find no significant polarization trend for Germany in our data. Overall, this finding highlights the added value of the longer time-series offered by our data.

Hence, the comparison between estimates should focus on the same period of analysis. Since we only work with post electoral data, the first data point in common with Boxell et al. (2021) is the 1980 election. The comparison should thus focus on the period 1980-2017. Another point worth considering has to do with the fact that the Politbarometer enables measurements of affective polarization in and out of election years. It has been clearly established that affective polarization increases as a function of the salience of elections (Hernandéz et al., 2021). Therefore, it is reasonable to assume that measurements from non-election years yield lower levels of affective polarization. And, in fact, when we compare Boxell et al.’s (2021) estimates from the Politbarometer for election years vs. non-election years, the former generally tend to yield higher affective polarization levels. Furthermore, post-electoral surveys tend to be fielded closely after the election. Hérnandez et al. (2021) found that voters’ affective polarization is sensitive to the distance in terms of days from the election day. If the Politbarometer fieldwork dates are further away from the election day than the German post-election survey, it may yield lower levels of affective polarization even in election years. This factor may also contribute to explain the discrepancy in the trends.

Based on the previous points, we give it that the focus of the comparison should thus be on the same period of analysis, and in the same election years (N=11). We have plotted a comparison of our estimates vs. Boxell et al.'s (2021) for those data points (Figure S5).

Figure S5. Comparison of estimates from our data and Boxell et al. (2021): 1980-2017

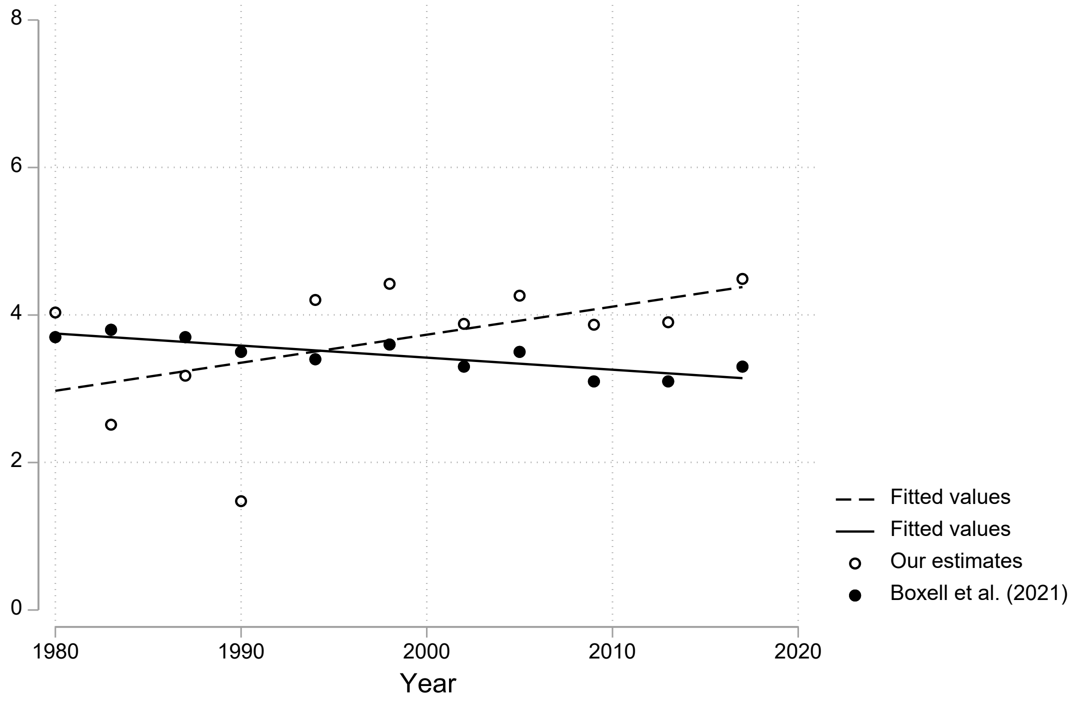

After the 1990 election, the trends from both datasets are almost perfectly symmetrical. This suggests a strong convergence between the two data sources, which should result in nearly identical trends over the same period. Nonetheless, our estimates are consistently higher than Boxell et al. (2021). Besides minor differences in the formulas which may be unaccounted for, this is, in all likelihood, owed to the fact that Boxell et al. (2021) only use data from West Germany, whereas our post-electoral data covers both West and East Germany. As is clearly demonstrated in Appendix Figure 11 from Boxell et al. (2021), polarization in East Germany is constantly higher than in West Germany. Therefore, by considering East Germany, we likely inflate our estimates compared to Boxell et al. (2021). However, it should be highlighted again that the trends for both the data sources unfold in parallel over this period. Also in the 1980 election, the estimates almost perfectly converge.

Nevertheless, in 1983, 1987 and 1990 there is a significant discrepancy between estimates from both datasets. Moreover, this difference goes in the opposite direction of all other data points, i.e., our estimates yield lower levels of AP than Boxell et al. (2021). It is, therefore, essential to understand what is behind this dissonance.

As per Table S4 of our manuscript, from 1961 to 1998, we rely on data harmonized by the European Voter project. For reasons unknown to us, in 1983, 1987, and 1990, the European Voter featured fewer feeling thermometers than those available in the German post-electoral survey. Therefore, our estimates for these years were calculated based on the CDU/CSU coalition, FDP and SPD for 1983 and 1987, to which PDS was added in 1990. The Politbarometer, instead, also included the Greens, in 1983 and 1987, as well as the Republikaner in 1990. For as low as the electoral size of these parties was back then, naturally impacting on the weighting of the respective feeling thermometer scores, these parties, also because ideologically more extreme, create the conditions for more affective polarization among the electorate.

We recognize that estimating affective polarization based on feeling thermometer scores for these three parties alone could be considered suboptimal, especially given that data for additional parties is available from other sources. Therefore, we have re-estimated affective polarization for these three elections using data from the German election studies (as we already did from 2002 onwards). This allows us to consider the same party supply Boxell et al. (2021) did using the Politbarometer. As demonstrated below, this approach corrects the original problem, producing estimates which are symmetrical to Boxell et al. (2021).

Figure S6. Comparison of estimates from Boxell et al. (2021) and alternative data for 1983-1990

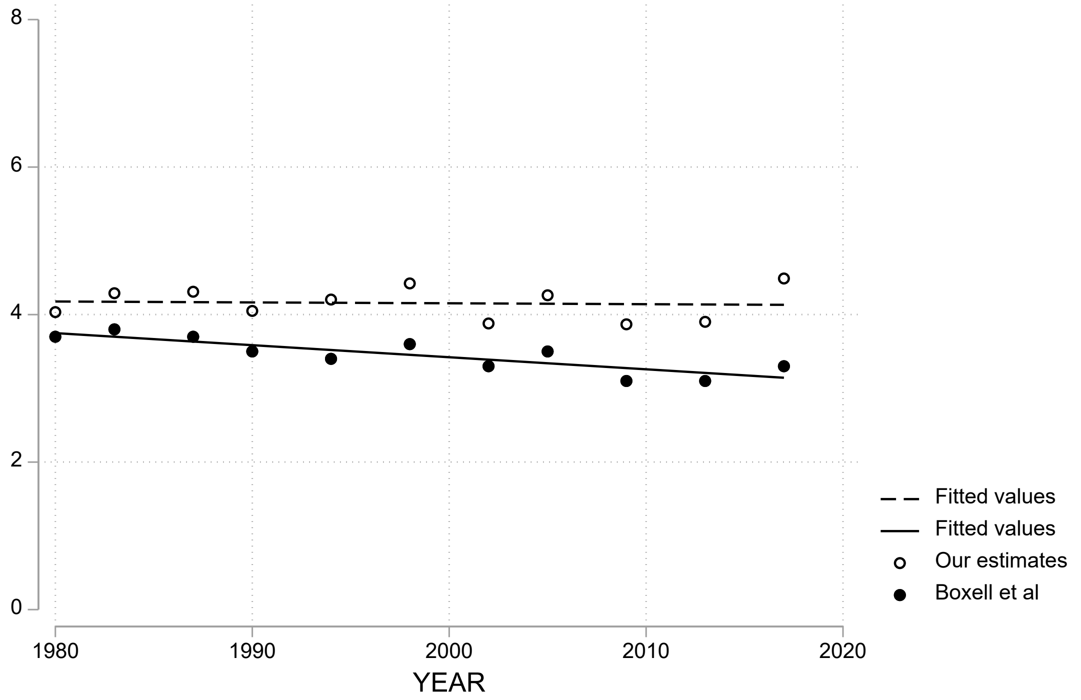

While this is certainly important to partially explain the divergences across trends, by all means the most important factor has to do with the length of the time series. As said earlier, once we zoomed in on the coincident period of analysis 1980-2017 no longer did we detect a significant polarization trend, even when using the original dissonant estimates from the European Voter. Hence, while changing the estimates from 1983, 1987, and 1990, does make our trends almost perfectly symmetrical to Boxell et al. (2021) between 1980-2017, it does not change the fact that, once we consider the whole period of analysis, we still uncover a significant polarization trend for Germany. Below are the results of the two OLS regression models using the original European Voter data (as per Table 1 of the manuscript) and the revised estimates for 1983, 1987, and 1990.

Table S5. Comparing OLS estimates for German data using the original data from the European Voter and three revised estimates for 1983, 1987, and 1990

	b	Adj. se	p-value	N
Original estimates (European Voter)	.025	.012	.039	16
Revised estimates for 1983, 1987, 1990	.023	.011	.034	16

Note: The *b* coefficients come from unstandardized bivariate linear regressions with affective polarization as the dependent variable and survey year as the independent variable. Adjusted standard errors and p-values are computed following Imbens and Kolesar (2016).

As clearly demonstrated in Table S5, the longitudinal trends are nearly identical. Since changing the data source does not alter the substantive interpretation of our findings for Germany, we preferred to keep the European Voter data for consistency reasons.
